# Monthly Summary

**Published:** 1870-04

**Authors:** 


### MONTHLY SUMMARY.

*Carbolic Acid.*—As a therapeutic agent, it has been most extensively used as an external application.

In sloughing wounds, a solution composed of one part acid to forty parts of water produces the most marvelous effects: it destroys all fetor, facilitates the separation of the slough, and causes parts beneath to assume a healthy appearance. It seems, also, to have the effect of promoting the growth of healthy granulations, and of hastening the healing process of wounds. It has been used successfully in several forms of skin diseases, *viz.*, lepra, tinea capitis, rupia, and eczema. It has proved a valuable agent in the treatment of hemorrhoidal affections and in fistula It is a valuable caustic, it only affecting a superficial layer of the tissue to which it is applied, hence its use would be indicated in diptheria and malignant sore throat. But carbolic acid has also been used internally, with beneficial results. One drop, given in the form of a pill, has checked vomiting when other remedies had failed to produce any effect.

It has been highly recommended in cases of dyspepsia, accompanied with pain in the stomach after eating.

It also has been largely used by many eminent French physicians in the treatment of phthisis. A large number of patients in different stages have been treated, with the most favorable results. The mode of administration is as follows:—"Fifteen drops of pure acid is dissolved in ʒ ij. of spirits, and the solution mixed with ℥ xxxij. of water; this quantity is administered daily, partly by the stomach and partly by the inhalation of fluids in a pulverized form."

Owing to the great demand for carbolic acid, it is largely adulterated. The article most commonly used for this purpose is coal-tar oil, but it can be easily detected.

Pure cabolic acid is soluble in from 20 to 60 parts of water, or twice its bulk of solution of caustic soda, while tar oil is nearly insoluble. Therefore, to test carbolic acid, we have only to put a drachm of it in a pint bottle, pour on it half a pint of warm water, and shake at intervals for half an hour, when the amount of oily matter will show the impurity. Or dissolve one part of caustic soda in ten parts of carbolic acid, the residue will show the amount of impurity.—*Dr. W. Little—Drug. Circ.*

---

*A Tooth Driven into the Antrum.*—A man was admitted to St. Mary's Hospital, who, three years and a half before, from a severe fall, had lost the right upper lateral incisor tooth, a few weeks after which an abscess formed, and discharged, both through the alveolus of the lost tooth, and through a small opening in the cheek. He had been treated by many naval surgeons, for what was supposed to be caries of the maxilla. The discharge from the alveolus had now ceased and the orifice healed over, but there was a constant profuse discharge from a sinus in the cheek, half an inch outside the nostrils, with considerable pain and swelling of the side of the face. On probing the sinus Mr Walton detected a smooth hard substance like tooth enamel, and after enlargening the opening, extracted with a forceps, in a perfect condition, the lost tooth, which was lying loose in the antrum. The pain and swelling subsided, the discharge ceased, and the wound soon healed.—*Med. Times & Gazette.*

*Action of Nitrate of Silver on Nervous Tissue.*—M. Grandry has communicated to the *Centralblatt*, the results of his observations on the action of nitrate of silver on nervous tissue. He used the tissue obtained from the frog and rabbit for his experiments, and placed portions of both from the centres and the nerves in a one-fourth per cent. solution, macerating them for five days in the dark, and then exposing them for three days to bright light. If the surface of the cord thus treated be carefully teazed out with needles, the axis-cylinders are found to exhibit a very regular and sharply-defined transverse striation—clear, unstained striæ, alternating with deeply-tinted ones. The breadth of the dark striæ varies from one to five thousandths of a millimetre, that of the clear, from one to three thousandths. In addition to the transverse striation, the axis-cylinder also exhibits a well-marked longitudinal striation, so that it presents a singularly close, though probably only superficial, analogy to a muscular fibre. Examination by polarized light, however, does not furnish any evidence of the existence of a doubly refractile substance. M. Grandry observed a similar transverse striation in the bodies of, and in the processes given off from, ganglion cells, especially in those of the anterior horn of the cervical portion of the spinal cord.—*Lancet.*

---

*Neuralgia and its Treatment by Electrization.*—In a paper contributed to the *Medical Record*, Drs. Rockwell and Beard give the results of their treatment of neuralgia by the Faradaic current. Of fifty cases, twenty-four recovered; eight approximately recovered; six were decidedly benefited; seven temporarily relieved; two not benefited, and three temporarily aggravated. Some of the cases were mild, but the majority had been vainly treated by medication. General electrization was employed in all cases, and is considered far preferable to the local application of the current. In some cases Faradaic electricity serves only to aggravate the pain; under such circumstances the galvanic current often proves serviceable. The instruments recommended are Dr. Jerome Kidder's, when Faradaization is desired, and Chester's air-tight galvanic apparatns, if the continuous current be preferable.—*Pacific Med. & Surg. Journal.*

*Indication of Longevity.*—H. P. Gatchell, M.D., Kenosha, Wis. (*The U. S. Medical and Surgical Journal*), reports a conversation with the late Dr. Beteley, of Cleveland, concerning the relation of the lobe of the ear, in which the latter informed Dr. G. that he had noticed in almost all old people, whom he had observed, long lobes to the ears.

Dr. Powell was accustomed to derive his indication of longevity from the angle subtended by a line drawn from the outer angle of the eye, through the orifice of the ear, and another drawn from the same point to the occipital tuberosity. But the angle in this case is determined by the depth of the occiput, and finds its explanation in Buchanan's doctrine of this region as that of animal force.

Dr. G. believes that the lobe of the ear is directly to the medulla oblongata, and only indirectly to the occiput. There is a general relation between the development of the occiput and the medulla.

The physician can assure the patient with long lobes, in the absence of malignant disease, of the probability of long life. He can also speak with more confidence in regard to recovery from either acute or chronic diseases, when the lobe is long than when it is short. Nor will he fail to observe that a large proportion of sickly people have a short lobe, or none at all.

Where a naturally strong constitution has suffered from excesses, the long lobe has become withered and wrinkled. And when one side of the brain, as indicated by a seated pain, has suffered more than the other, he will find the lobe of that side more withered than the other. Whatever tends to enfeeble the constitution, whether excessive toil, study, or venery, contributes to that change in the lobe.—*Med. Record.*

---

*Green Line on the Gum from Copper Poisoning.*—Dr. Donald Frazer (*Canada Journal of Dental Sciences*) records a case of poisoning from copper occurring in a sailor just returned from a long voyage. The green line on the gum was analogous to the blue line from lead poisoning, and was distinctly visible, having been received through the medium of lime-juice which had been left in a copper vessel.

*Deaths from Chloroform.*—During the year we have recorded in this journal twenty-five cases of death from chloroform. Commentary upon these figures is unnecessary. Remembering the comparatively insignificant number of alleged deaths from the inhalation of ether recorded since its introduction to the present time, and that there is not one of these "which cannot be explained on some other ground equally plausible" (Rep. of Ether Comm. of Bost. Soc. for Med. Improvement; Extracts from *Record*, vol iv., Supplement, p. 216)—a statement undoubtedly not true as regards chloroform—we must indorse Prof. Stille's remark (Mat. Med. and Therap., vol. ii. p. 115, 3d ed.) that "the surgeon who employs it [chloroform] assumes a responsibility of life and death for which neither his office nor the moral law afford him any license."—*Medical News and Library.*

---

*Blackened Teeth from Tea.*—We were lately consulted by a lady on account of discoloration of her teeth, which she supposed might be owing to some pills that we had prescribed for her. On investigation, the effect was traced to the tea used at the boarding house, which was kept from day to day in a tinned vessel, and heated up at meal-times, with the addition of a fresh supply of the material. The tin having worn off, left a surface of iron, and the infusion, in cooling, acted chemically on the iron, making a tannate or gallate of iron. The boarders had been regaling themselves on *ink!* We are told that this is quite a common custom at boarding houses and restaurants.—*Pacific Med. and Surg. Journal.*

---

*Chloroform in Infantile Convulsions.*—There is no speedier relief for these troublesome adjuncts to childhood than chloroform, given in doses from gtts. x to xxx in cold water, care being taken that the chloroform be intimately mingled with the liquid, as it forms at last but a mechanical mixture. It is also a very efficient remedy in cases of uncontrolable vomiting, or retching, caused by spasmodic action of the muscular coats of the stomach in adults.—*Oregon Med. & Surg. Reporter.*

